# Targeting Soluble Amyloid Oligomers in Alzheimer’s Disease: A Hypothetical Model Study Comparing Intrathecal Pseudodelivery of mAbs Against Intravenous Administration

**DOI:** 10.3390/diseases13010017

**Published:** 2025-01-16

**Authors:** Manuel Menendez-Gonzalez

**Affiliations:** 1 Departamento de Medicina, Facultad de Ciencias de la Salud, Universidad de Oviedo, ES-33006 Oviedo, Spain; menendezgmanuel@uniovi.es; 2 Servicio de Neurología, Hospital Universitario Central de Asturias, ES-33011 Oviedo, Spain; 3 Instituto de Investigación Sanitaria del Principado de Asturias (ISPA), ES-33011 Oviedo, Spain

**Keywords:** intrathecal pseudodelivery, soluble amyloid-β (Aβ), monoclonal antibodies (mAb), amyloid PET scan

## Abstract

Background/Objective: Neurotoxic soluble amyloid-β (Aβ) oligomers are key drivers of Alzheimer’s pathology, with evidence suggesting that early targeting of these soluble forms may slow disease progression. Traditional intravenous (IV) monoclonal antibodies (mAbs) face challenges, including limited brain penetration and risks such as amyloid-related imaging abnormalities (ARIA). This hypothetical study aimed to model amyloid dynamics in early-to-moderate Alzheimer’s disease (AD) and compare the efficacy of IV mAn with intrathecal pseudodelivery, a novel method that confines mAbs in a subcutaneous reservoir for selective amyloid clearance in cerebrospinal fluid (CSF) without systemic exposure. Methods: A mathematical framework was employed to simulate Aβ dynamics in patients with early-to-moderate AD. Two therapeutic approaches were compared: IV mAb and intrathecal pseudodelivery of mAb. The model incorporated amyloid kinetics, mAb affinity, protofibril size, and therapy-induced clearance rates to evaluate the impact of both methods on amyloid reduction, PET negativity timelines, and the risk of ARIA. Results: Intrathecal pseudodelivery significantly accelerated Aβ clearance compared to IV administration, achieving amyloid PET scan negativity by month 132, as opposed to month 150 with IV mAb. This method demonstrated no ARIA risk and reduced amyloid reaccumulation. By targeting soluble Aβ species more effectively, intrathecal pseudodelivery emerged as a safer and more efficient strategy for early AD intervention. Conclusions: Intrathecal pseudodelivery offers a promising alternative to IV mAbs, overcoming challenges associated with blood–brain barrier penetration and systemic side effects. Further research should focus on optimizing this approach and exploring combination therapies to enhance clinical outcomes in AD.

## 1. Introduction

Alzheimer’s disease (AD) is a progressive neurodegenerative disorder characterized by the accumulation of β-amyloid (Aβ) peptides in the brain, forming extracellular plaques that disrupt neuronal function and contribute to cognitive decline. The amyloid hypothesis, first proposed in the early 1990s, posits that Aβ aggregation initiates a cascade of neurodegenerative processes leading to AD pathology [1]. Despite advances in understanding, many therapies targeting Aβ have shown limited success in clinical outcomes, highlighting the complexity of Aβ species and their contributions to disease progression. In recent years, it became clear the key pathogenic role soluble oligomers and protofibrils play in the disease [2,3,4,5,6], thus becoming an evident target. Even when total CSF Aβ is low in symptomatic AD, CSF Aβ oligomers peak in early stages of AD preceding tau pathology [2]. Indeed, quantification of Aβ oligomers in blood showed impaired clearance from brain in ApoE E4 positive subjects [3]

Clinical biomarkers such as cerebrospinal fluid (CSF) Aβ42 levels and positron emission tomography (PET) imaging for amyloid offer critical insights into disease progression and treatment effects [7,8,9]. However, discordance between CSF and PET biomarkers underscores the heterogeneity in soluble and aggregated Aβ species across different disease stages [10].

Monoclonal antibodies (mAbs), such as Donanemab, Gantenerumab, Aducanumab, and Lecanemab, have demonstrated the ability to reduce amyloid burden through immune-mediated mechanisms. Each of these antibodies targets distinct species of Aβ: Donanemab preferentially binds to N-terminally truncated Aβ species, such as pyroglutamate-Aβ, which are prevalent in aggregated plaques; Gantenerumab targets both soluble and fibrillar Aβ, showing affinity for aggregated plaques and protofibrils; Aducanumab selectively binds aggregated Aβ, primarily targeting fibrillar species; and Lecanemab demonstrates a unique affinity for protofibrils, intermediate forms between soluble oligomers and fibrillar plaques. Despite these advances, their limited capacity to cross the blood–brain barrier (BBB) and associated risks, such as amyloid-related imaging abnormalities (ARIA), have hindered their clinical success. Furthermore, these therapies exhibit limited efficacy in addressing soluble Aβ oligomers, which are increasingly recognized as critical drivers of early AD pathology. This limitation underscores the pressing need for alternative strategies that selectively target soluble Aβ species while minimizing systemic exposure and reducing associated risks [11,12,13,14,15,16,17,18].

A promising candidate addressing this gap is Sabirnetug (ACU193), a humanized IgG2 monoclonal antibody specifically designed to target soluble Aβ oligomers. These oligomers are considered the most neurotoxic forms of Aβ, implicated in neuronal death and memory loss. Developed through a collaboration between Acumen and Merck between 2004 and 2011, Sabirnetug has demonstrated high specificity and efficacy in preclinical studies, effectively blocking oligomer interactions with hippocampal neurons, reducing plaque deposition, and improving behavioral outcomes in mouse models of AD. Pharmacokinetic studies revealed that ACU193 achieved brain concentrations far exceeding the levels of Aβ oligomers typically found in Alzheimer’s CSF. Beyond therapeutic applications, Sabirnetug has also been employed experimentally for detecting Aβ oligomers using imaging and immunoassays [19,20,21,22,23]. In its early clinical development, Phase 1 trials involving individuals with mild cognitive impairment (MCI) or mild AD demonstrated that Sabirnetug achieved dose-dependent CSF concentrations, significant target engagement, and a modest reduction in plaque load. While some cases of ARIA were reported, they were successfully resolved. Following these findings, the ALTITUDE-AD study was initiated in 2024 as a Phase 2/3 trial to evaluate the effects of monthly Sabirnetug doses on cognitive and functional outcomes in early AD. Although enrollment has been scaled down, the study is expected to conclude in 2026 [19,20,21,22,23]. In late 2024, a Phase 1 trial comparing intravenous and subcutaneous administration was performed. This trial incorporated rHuPH20, an enzyme designed to enhance absorption, underscoring ongoing efforts to refine delivery methods.

To address the challenges posed by BBB penetration, alternative strategies such as receptor-binding molecules and intrathecal pseudodelivery systems have emerged. For instance, Trontinemab, a bispecific fusion protein, combines Gantenerumab with a human transferrin receptor-targeting molecule to enhance brain delivery. These innovative approaches underscore the growing focus on optimizing CNS-targeted therapies for AD [24].

An alternative to the systemic administration of drugs to bypass the limit of BBB penetration is intrathecal pseudodelivery. The mechanism of action of this route is rooted in the CSF-sink therapeutic strategy. The CSF-sink mechanism facilitated by intrathecal pseudodelivery accelerates amyloid clearance from the brain by maintaining an equilibrium between CSF and interstitial fluid (ISF) amyloid pools [25,26]. Preclinical studies demonstrate that this method achieves rapid and targeted reductions in soluble Aβ, particularly during early disease stages where soluble species predominate [25,26]. Compared to IV administration, intrathecal delivery offers enhanced safety, avoiding infusion-related reactions and systemic side effects while maintaining therapeutic efficacy.

This study builds upon these findings, employing a continuous β-amyloid CSF/PET imbalance model to assess the dynamics of Aβ aggregation and evaluate the impact of IV and intrathecal pseudodelivery of a mAb against soluble Aβ—such as Sabirnetug—on PET and CSF Aβ pools, focusing on both soluble and fibrillar species. We model Aβ dynamics to compare the efficacy of IV and intrathecal pseudodelivery of Sabirnetug in clearing amyloid from the brain. By integrating data on amyloid kinetics, mAb affinity, protofibril size, and dosage requirements, this analysis predicts the therapeutic impact of these approaches on amyloid clearance and the time to achieve amyloid PET scan negativity. Our findings aim to inform the optimization of personalized treatment strategies for AD.

## 2. Materials and Methods

### 2.1. Study Design

This hypothetical study employed a mathematical modeling framework to simulate the dynamics of Aβ over time, focusing on differences between soluble CSF and aggregated PET Aβ pools. We utilized a continuous imbalance model inspired by Mastenbroek et al. (2024) [10], which provides a robust way to capture AD heterogeneity. The model integrates an Aβ-aggregation score derived from CSF-Aβ42 and global Aβ-PET data [27], characterizing the imbalance between soluble and fibrillar Aβ species across disease stages. This score was further refined to capture the distinct impacts of IV and intrathecal pseudodelivery therapies on amyloid dynamics. 

### 2.2. Patient Populations

We modeled two hypothetical patient cohorts reflecting typical AD pathology:

1. Intravenous mAb cohort: Representing patients receiving monthly IV infusions of a mAb targeting soluble Aβ oligomers [19,20,21,22,23].
2. Intrathecal pseudodelivery cohort: Representing patients receiving continuous subcutaneous reservoir-based delivery of mAbs targeting soluble Aβ in CSF [25,26,28,29].

Both cohorts were calibrated to represent disease onset at 120 months, with a PET positivity threshold set at 35 centiloids, marking the beginning of symptomatic AD [14,15]. Patient populations were modeled to account for differences in CSF/PET dynamics, reflecting the distinct therapeutic impact of soluble and fibrillar amyloid clearance.

### 2.3. Equations for Aβ Dynamics

A detailed description of the equations can be found in Supplementary Materials. In summary, the equations for Aβ dynamics are as shown below.

#### 2.3.1. Intrathecal Pseudodelivery Equation

$$A(t)=\left(A_{0}-\frac{P}{C+C_{IT}}\right)e^{-\left(C+C_{IT}\right)t}+\frac{P}{C+C_{IT}}$$

where

- *A*(*t*): soluble Aβ concentration in the CSF at time *t* (pg/mL).
- *A*_0_ = 100 pg/mL: initial concentration of soluble Aβ at baseline.
- *P* = 180 pg/mL/month: constant production rate of soluble Aβ.
- *C* = 0.05 month^−1^: natural clearance rate of soluble Aβ.
- *CIT* = 0.90 month^−1^: therapy-induced clearance rate for intrathecal pseudodelivery [28].

#### 2.3.2. Intravenous (IV) mAb Equation

$$A(t)=\left(A\left(t_{n}^{+}\right)-\frac{P}{C}\right)e^{-C\left(t-t_{n}\right)}+\frac{P}{C}$$

where

- *A*(*t*): soluble Aβ concentration in the CSF at time *t* (pg/mL).
- *A*(*t* + *n*): soluble Aβ concentration immediately after the *n*-th dose (pg/mL).
- *P* = 180 pg/mL/month: constant production rate of soluble Aβ.
- *C* = 0.05 month^−1^: natural clearance rate of soluble Aβ.
- *E* = 0.60: fractional reduction in soluble Aβ per IV dose [19,20,21,22,23].

### 2.4. Simulation Metrics

Primary outcomes in simulation metrics included the following:

1. Time to PET negativity (Aβ burden below 24 centiloids).
2. Magnitude of CSF-Aβ reduction over time.
3. Risk of amyloid reaccumulation upon therapy discontinuation.

## 3. Results

### 3.1. Amyloid Clearance Dynamics

**Figure 1:**
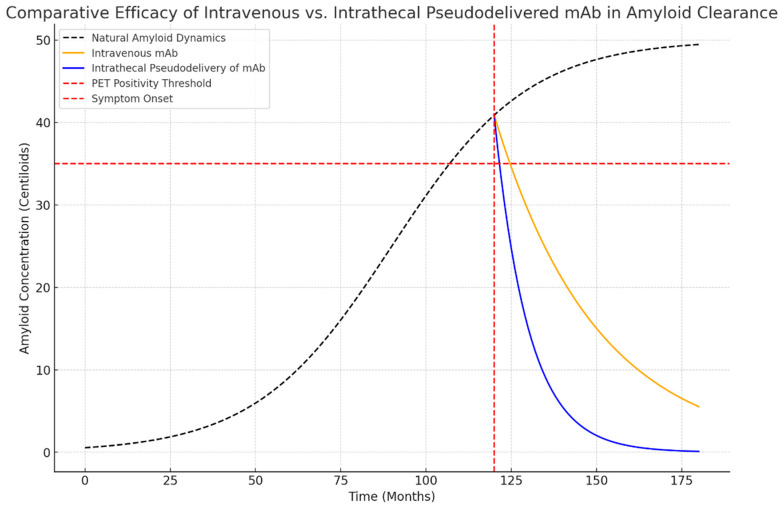
Simulated dynamics of amyloid clearance using intravenous (IV) and intrathecal pseudodelivery of monoclonal antibodies (mAbs). Intrathecal pseudodelivery achieves rapid reduction of soluble Aβ in cerebrospinal fluid (CSF), leading to amyloid PET negativity by month 132. In contrast, intravenous administration reduces soluble Aβ more gradually, achieving PET negativity by month 150.

Simulation results revealed distinct differences in the therapeutic impact of IV and intrathecal pseudodelivery of mAb (Figure 1):

1. **Time to PET Negativity:**
  ○ Intrathecal pseudodelivery of mAb achieved PET negativity at approximately 132 months.
  ○ IV mAb reached PET negativity at 150 months.
2. **CSF-Aβ Reduction:**
  ○ Intrathecal pseudodelivery of mAb led to an accelerated reduction in soluble Aβ, achieving a 90% decrease in CSF-Aβ levels within 12 months.
  ○ IV mAb showed a slower decline, with a 60% reduction over the same period.

### 3.2. Long-Term Effects

1. **Amyloid Reaccumulation:**
  ○ Upon discontinuation, amyloid reaccumulated within 12–18 months in both cohorts, but intrathecal pseudodelivery delayed PET positivity by an additional 6 months compared to IV therapy.

## 4. Discussion

Soluble Aβ oligomers are strongly linked to early cognitive decline and represent a critical therapeutic target [2,3,4,5,6,27]. Intrathecal mAb pseudodelivery achieves rapid clearance of these neurotoxic species, potentially delaying cognitive symptom onset. This approach offers a theoretical advantage over traditional IV mAbs, which are hindered by limited BBB penetration and systemic side effects. However, as this study is based on a mathematical model, it should be considered hypothesis-generating rather than conclusive.

Our findings align with clinical observations showing amyloid reaccumulation within 12–18 months after therapy discontinuation, with PET positivity returning within 18–24 months [30]. These data highlight the need for sustained or long-term intermittent therapeutic regimens to maintain amyloid clearance and stabilize cognitive function. The β-amyloid CSF/PET modeling framework used in this study provides insights into how different amyloid species contribute to AD progression and therapeutic response [7,8,9,10,27]. By focusing on soluble Aβ, predominant in early AD, intrathecal pseudodelivery demonstrated faster and more targeted amyloid clearance compared to IV mAbs (Table 1). This approach utilizes the CSF sink mechanism to enhance soluble amyloid clearance, avoiding systemic side effects and ARIA risks—significant limitations of IV therapies. Conversely, IV mAbs, which better target aggregated amyloid species detectable on PET scans, may be more effective for later-stage AD when fibrillar amyloid pathology dominates (Table 1).

Intrathecal pseudodelivery, however, has its own challenges. Stability of mAbs in the subcutaneous reservoir is a key concern, as prolonged exposure may lead to degradation or aggregation, reducing efficacy. Addressing these issues will require the development of stabilizers and optimized redosing intervals to ensure long-term functionality. Additionally, while the theoretical safety profile of intrathecal pseudodelivery is promising, the absence of experimental clinical validation remains a significant limitation.

Another limitation is the underlying hypothesis that extracellular Aβ oligomers are the primary toxic species in AD. Alternative theories suggest intraneuronal Aβ oligomers or tau-mediated mechanisms may play a relevant role. If these pathways predominate, intrathecal pseudodelivery, which primarily targets extracellular compartments, may have limited therapeutic impact. This underscores the need for complementary strategies targeting both extracellular and intracellular toxic species, potentially through small-molecule inhibitors or gene therapies.

Patient acceptability is another critical factor. Although the intrathecal catheter and subcutaneous reservoir are only moderately invasive, concerns about procedural safety and maintenance may impact adherence. However, contrary to some preconceived ideas, relatives of elderly patients are often in favor of a high level of intervention, even in the presence of cognitive impairment [31]. Clear communication about the benefits and risks, supported by robust clinical trial data, will be essential to improve patient and caregiver confidence in this approach.

Future research must address several critical aspects to advance intrathecal pseudodelivery as a therapeutic approach for AD. Enhancing the durability and biocompatibility of reservoir materials is essential, as these factors directly impact the stability and functionality of monoclonal antibodies in the subcutaneous reservoir. Additionally, optimizing redosing intervals to prevent antibody degradation and maintain consistent therapeutic delivery over time will be pivotal for clinical translation.

Exploring combination therapies is also important, particularly those integrating intrathecal pseudodelivery with strategies addressing other targets to maximize the advantage of combining diverse immunotherapies conveniently under one safe route.. This dual approach could maximize efficacy across different disease stages by addressing both soluble and fibrillar amyloid species [32]. 

Validation through rigorous experimental and clinical studies of intrathecal pseudodelivery remains a top priority. Such studies should not only confirm safety and efficacy but also offer insights into its practical implementation, including patient-centered concerns. Furthermore, complementary strategies targeting intraneuronal amyloid and other pathogenic mechanisms, such as tau, should be investigated to develop a more comprehensive therapeutic framework. Collectively, these efforts will be essential for unlocking the full potential of intrathecal pseudodelivery and expanding its role in AD treatment.

## 5. Conclusions

Intrathecal pseudodelivery of mAbs targeting soluble Aβ represents a novel and potentially transformative approach to AD treatment. This modeling study demonstrates its ability to accelerate amyloid clearance, delay cognitive decline, and mitigate risks such as ARIA, which are associated with intravenous mAbs. These findings suggest that intrathecal delivery could provide a safer and more efficient strategy for early intervention in AD.

However, this approach has limitations, including its inability to target insoluble plaques and the need for further optimization of the reservoir system. Combining intrathecal delivery with plaque-targeting therapies could offer a more comprehensive treatment strategy. Future research should focus on addressing technical challenges, validating long-term efficacy, and exploring patient-centered solutions to improve acceptability and outcomes.

This study provides a theoretical framework to guide future investigations, emphasizing the potential of personalized strategies to enhance safety and efficacy in AD management.

## Figures and Tables

**Table 1:** Comparative table of intravenous delivery of mAb against intrathecal pseudodelivery of mAb.

Delivery Method	Target	Efficacy	Safety	Invasiveness	Key Limitations
IV mAB	Soluble and insoluble Aβ	Gradual clearance; slower PET negativity	High ARIA risk (up to 30%); systemic side effects	Minimally invasive (IV infusions)	Limited BBB penetration; ARIA risk
Intrathecal pseudodelivery of mAB	Soluble Aβ	Rapid clearance; faster PET negativity	No ARIA risk; no systemic side effects	Moderately invasive (subcutaneous reservoir)	Does not target insoluble plaques; stability challenges

## Data Availability

No new data was generated in this study.

## Supplementary Materials

The following supporting information can be downloaded at: https://www.mdpi.com/article/10.3390/diseases13010017/s1, Supplementary Materials shows the elaboration of equations, assumptions, and parameters used in the mathematical model, along with their justifications.
