# Supplementary material for: Targeting Soluble Amyloid Oligomers in Alzheimer’s Disease: A Hypothetical Model Study Comparing Intrathecal Pseudodelivery of mAbs Against Intravenous Administration
Source: Diseases. 2025 Jan 16;13(1):17. doi: 10.3390/diseases13010017 (PMC11764272; doi:10.3390/diseases13010017)
Supplement: Supplementary file 1 [file diseases-13-00017-s001.zip › diseases-3347423-supplementary.pdf]

Supplementary Materials: Equations, assumptions and parameters used in the mathematical model and their justifications

A. Step-by-step derivation of equations

A.1. Intrathecal pseudodelivery model

The dynamics of soluble Aβ in the CSF during intrathecal pseudodelivery are governed by a first-order differential equation incorporating production, natural clearance, and therapy-induced clearance. The equation can be written as:

$$\frac{dA(t)}{dt} = P - (C + C_{IT})A(t)$$

Where:

- A(t): Soluble Aβ concentration in the CSF at time ttt (pg/mL).
- P: Constant production rate of soluble Aβ (pg/mL/month).
- C: Natural clearance rate of soluble Aβ (month−1).
- CIT: Therapy-induced clearance rate for intrathecal delivery (month−1).

This is a linear first-order differential equation. Solving this equation yields:

$$A(t) = \left(A_0 - \frac{P}{C + C_{IT}}\right) e^{-(C+C_{IT})t} + \frac{P}{C + C_{IT}}$$

Where:

- A0: Initial concentration of soluble Aβ (pg/mL).

Derivation steps:

1. **Start with the general solution** for a first-order linear differential equation:

$$\frac{dA}{dt} + kA = b$$

Where

$k = C + C_{IT}$ and $b = P$

2. **Integrate the equation:**

$$A(t) = Ce^{-kt} + \frac{b}{k}$$

Where *C* is an integration constant determined by initial conditions.

3. **Apply the initial condition *A(0)=A0***

$$A_0 = C + \frac{P}{C + C_{IT}}$$

Solving for C, we get:

$$C = A_0 - \frac{P}{C + C_{IT}}$$

4. **Substitute back into the solution**:

$$A(t) = \left(A_0 - \frac{P}{C + C_{IT}}\right) e^{-(C+C_{IT})t} + \frac{P}{C + C_{IT}}$$

A.2. Intravenous Model

The dynamics of soluble Aβ during intravenous therapy account for periodic administration of the monoclonal antibody and the corresponding fractional reduction of Aβ concentration with each dose. The model is given by:

$$A(t) = \left(A(t_n^+) - \frac{P}{C}\right) e^{-C(t-t_n)} + \frac{P}{C}$$

Where:

- A(tn+): Soluble Aβ concentration immediately after the n-th dose.
- E: Fractional reduction per dose $(0 \leq E \leq 1)$

Derivation steps:

1. **Define dynamics between doses:** Between doses, the Aβ concentration follows the natural clearance dynamics:

$$\frac{dA}{dt} = P - CA(t)$$

2. **Solve the differential equation:** Using the same method as above, the solution is:

$$A(t) = \left(A(t_n^+) - \frac{P}{C}\right) e^{-C(t-t_n)} + \frac{P}{C}$$

3. **Incorporate the effect of dosing:** At each dosing time tnt, the soluble Aβ concentration is reduced by a fraction *E*:

$$A(t_n^+) = (1 - E)A(t_n^-)$$

Where *A(tn−)* is the concentration immediately before the dose.

4. **Combine dynamics into a piecewise model:** After the n-th dose, the concentration evolves as:

$$A(t) = \left(A(t_n^+) - \frac{P}{C}\right) e^{-C(t-t_n)} + \frac{P}{C}$$

B. Justification for assumptions

1. **Linear Dynamics:** The assumption of first-order dynamics for Aβ clearance is supported by prior studies of amyloid kinetics.
2. **Constant Production Rate:** The production rate *P* is assumed constant over time, reflecting stable Aβ generation in AD patients.
3. **Clearance Rates:** Therapy-induced clearance (*CITC*) is modeled as additive to natural clearance (*C*), consistent with the mechanism of action of monoclonal antibodies targeting soluble Aβ.
4. **Fractional Reduction (IV Model):** The fractional reduction *E* represents the efficacy of mAb dosing, derived from clinical trial data of similar therapies.
5. **PET Positivity Threshold:** The threshold for PET positivity (35 centiloids) can be considered relatively high while aligning with clinical definitions of amyloid positivity in symptomatic AD.

C. Parameters used in the mathematical nodel and their justifications

The parameters included in the equations for modeling the dynamics of soluble Aβ concentration in CSF during intrathecal pseudodelivery and intravenous mAb administration were chosen based on clinical, physiological, and experimental data. Here is a detailed explanation and justification for each parameter:

C.1. A(t): Soluble Aβ concentration in the CSF at time t (pg/mL)

Definition:
Represents the concentration of soluble Aβ in the CSF at any given time *t*, which is the key variable being modeled.

Justification:
Soluble Aβ in the CSF reflects the dynamics of Aβ production, clearance, and therapeutic effects. This parameter is directly measurable via CSF sampling in clinical trials, making it a practical and clinically relevant output metric.

C.2. *P*: Constant production rate of soluble Aβ (pg/mL/month)

Definition:
The rate at which soluble Aβ is produced in the central nervous system and released into the CSF.

Value Used:
P=180pg/mL/month

Justification:

- This value is derived from experimental studies on Aβ metabolism in healthy individuals and patients with Alzheimer's disease (AD).
- Studies have shown relatively stable production rates of Aβ, which allows this parameter to be modeled as a constant.
- References: Data from cerebrospinal fluid biomarker studies and experimental models.

C.3. *C*: Natural clearance rate of soluble Aβ (month−1)

Definition:
Represents the baseline rate at which soluble Aβ is naturally cleared from the CSF without therapeutic intervention.

Value Used:
C=0.05 month−1

Justification:

- The clearance of Aβ from the CSF occurs through multiple mechanisms, including drainage into the peripheral circulation and enzymatic degradation.
- The value of 0.05 month−1 aligns with observed Aβ turnover rates in longitudinal studies.
- This parameter accounts for age-related declines in clearance efficiency, which is particularly relevant in Alzheimer's disease.
- References: Natural clearance rates estimated from studies on Aβ kinetics.

C.4. *CIT:* Therapy-induced clearance rate for intrathecal pseudodelivery (month−1)

Definition:
The additional clearance rate of soluble Aβ induced by the intrathecal pseudodelivery of mAb.

Value Used:
CIT=0.90 month−1

Justification:

- This value represents the therapeutic impact of intrathecal pseudodelivery, which achieves higher exposure of mAb in the CSF compared to intravenous delivery.

- The higher clearance rate is supported by preclinical and early-phase clinical trial data demonstrating rapid binding and neutralization of soluble Aβ in the CSF when using intrathecal delivery systems.
- References: Pharmacokinetic studies and modeling data from experimental therapies targeting Aβ in CSF.

Model Refinement

While the current model provides valuable insights into the therapeutic potential of intrathecal pseudodelivery, incorporating additional biophysical parameters could significantly enhance its predictive accuracy. Key factors such as antibody-target interactions, binding kinetics, and physiological parameters warrant further exploration to improve the model's fidelity and applicability.

Antibody-Target Interactions

Modeling antigen-antibody dynamics using established kinetic equations, such as:

$$\frac{d[A]}{dt} = k_{\text{on}}[A\beta][pAb] - k_{\text{off}}[Complex]$$

provide a more detailed understanding of the binding and dissociation rates (konk_{\text{on}}kon and koffk_{\text{off}}koff) between monoclonal antibodies (pAbs) and soluble Aβ species. Experimentally determined dissociation constants (KDK_DKD) for pAb interactions with Aβ oligomers and fibrils should also be integrated to differentiate therapeutic efficacy across disease stages.

Physiological Considerations

Incorporating physiological parameters such as cerebrospinal fluid (CSF) flow rate (~0.3 mL/min), baseline Aβ concentration (~500 pg/mL), and the Aβ half-life in CSF (approximately 2–6 hours) could further refine predictions regarding antibody distribution and clearance. These parameters directly influence the equilibrium between free and antibody-bound Aβ, as well as the therapeutic dosing frequency required to sustain efficacy.

Model Challenges and Applications

The stability of monoclonal antibodies within the subcutaneous reservoir, particularly the risks of degradation or aggregation, should also be parameterized within the model. This could guide the optimization of reservoir materials and dosing intervals to enhance long-term functionality. Additionally, the integration of these dynamic interactions into the model would allow for better predictions of therapeutic outcomes and stage-specific interventions.

By addressing these aspects, the refined model could serve as a robust tool for evaluating the efficacy of intrathecal pseudodelivery and for designing combination strategies targeting both soluble and insoluble Aβ species.

Potential Impact on Equations

Incorporating these elements would not necessarily alter the equations you currently use but would expand them. For example:

1. **Binding Kinetics**:
   Adding konk_{\text{on}}kon and koffk_{\text{off}}koff terms to describe antibody-Aβ interactions would expand the model by explicitly accounting for dynamic binding and unbinding processes.

2. **CSF Flow Rate**:
   A term for flow rate (FCSFF_{\text{CSF}}FCSF) might be introduced to simulate antibody and Aβ distribution in the CSF compartment:

$$\frac{d[A]}{dt} = k_{\text{on}}[A\beta][pAb] - k_{\text{off}}[Complex] - F_{\text{CSF}}[A]$$

3. **Clearance Rates**:
   Incorporating Aβ half-life (t1/2t_{1/2}t1/2) could involve exponential decay terms:

$$\frac{d[A]}{dt} = \cdots - \frac{[A]}{\tau}$$

where $\tau = \ln(2)/t_{1/2}$.

Summary of parameters and justification

**Parameter**	**Value**	**Justification**
A(t)	Variable	Represents the output metric directly measurable in clinical trials.
P	180 pg/mL/month	Based on stable Aβ production rates observed in experimental and clinical studies.
C	0.05 month−1	Reflects natural Aβ clearance, validated by studies on CSF turnover and Aβ dynamics.
CIT	0.90 month−1	Reflects therapy-enhanced clearance via intrathecal delivery, supported by pharmacokinetic modeling.
k_on	Experimental value	Describes the rate of binding between Aβ species and monoclonal antibodies (pAb), informed by antigen-antibody interaction studies and experimental binding kinetics.
k_off	Experimental value	Reflects the rate of dissociation of Aβ-pAb complexes, critical for modeling antibody dynamics and therapeutic efficacy.
K_D	Experimental value	The dissociation constant provides insight into the binding affinity of pAbs for Aβ oligomers and fibrils, differentiating therapeutic effects across disease stages.
F_CSF	0.3 mL/min	Represents the CSF flow rate, which affects antibody distribution and Aβ clearance within the intrathecal compartment, based on physiological studies.
Aβ Half-Life (t1/2)	2–6 hours	Reflects the time required for half of the soluble Aβ in the CSF to be naturally cleared, based on experimental data on Aβ dynamics.
Antibody Stability	Experimental value	Accounts for potential degradation or aggregation of monoclonal antibodies in the subcutaneous reservoir, informed by experimental stability tests and material compatibility studies.

These parameters and their values ensure that the model captures the key physiological and therapeutic dynamics of soluble Aβ in the CSF during both natural and treatment-modulated scenarios. Each parameter has been chosen to provide realistic and interpretable predictions aligned with experimental and clinical data.

References

1. Bianchi, S.; Landi, G.; Marella, C.; Tesi, M.C.; Testa, C.; on behalf of the Alzheimer's Disease Neuroimaging Initiative. A Network-Based Study of the Dynamics of Aβ and τ Proteins in Alzheimer's Disease. Math. Comput. Appl. 2024, 29, 113. https://doi.org/10.3390/mca29060113
2. Mastenbroek SE, Sala A, et al. Continuous β-Amyloid CSF/PET Imbalance Model to Capture Alzheimer Disease Heterogeneity. Neurology. 2024.
3. Hardy J, Higgins GA. Alzheimer's disease: The amyloid cascade hypothesis. Science. 1992;256(5054):184-185.
4. Jack CR, et al. Tracking pathophysiological processes in Alzheimer's disease. Lancet Neurology. 2013;12(2):207-216.
5. Krafft GA, Jerecic J. ACU193: An Immunotherapeutic Poised to Test the Amyloid β Oligomer Hypothesis. Front Neurosci. 2022;16:848215.
6. Coto Vilcapoma MA. Nanoféresis inmunoselectiva de Aβ en el líquido cefalorraquídeo como tratamiento de la enfermedad de Alzheimer (Doctoral thesis) https://hdl.handle.net/10651/75843
7. Ashton NJ, Brum WS, Di Molfetta G, Benedet AL, Arslan B, Jonaitis E, et al. Diagnostic Accuracy of a Plasma Phosphorylated Tau 217 Immunoassay for Alzheimer Disease Pathology. JAMA Neurol. 2024;81(3):255-263. doi: 10.1001/jamaneurol.2023.5319.
8. Collij LE, Bollack A, La Joie R, Shekari M, Bullich S, Roé-Vellvé N, Koglin N, Jovalekic A, Garciá DV, Drzezga A, Garibotto V, Stephens AW, Battle M, Buckley C, Barkhof F, Farrar G, Gispert JD; AMYPAD consortium. Centiloid recommendations for clinical context-of-use from the AMYPAD consortium. Alzheimers Dement. 2024 Dec;20(12):9037-9048. doi: 10.1002/alz.14336. Epub 2024 Nov 20. PMID: 39564918; PMCID: PMC11667534.
